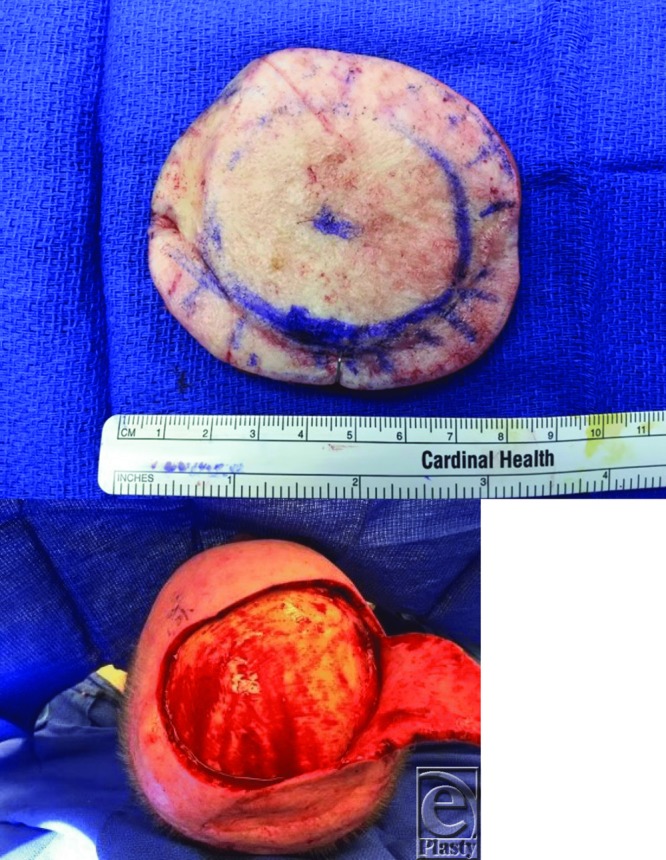# Desmoplastic Melanoma

**Published:** 2017-09-07

**Authors:** Anthony Maurice Kordahi, Joshua B. Elston, Ellen M. Robertson, C. Wayne Cruse

**Affiliations:** Division of Plastic Surgery, Department of Surgery, University of California San Diego; and University of South Florida Morsani College of Medicine, Tampa

**Keywords:** desmoplastic melanoma, skin cancer, spindle cell, neurotropism, desmoplasia

## DESCRIPTION

A 69-year-old man presented with a 2-year history of a recurrent, nonpigmented, nodular scalp lesion. He underwent surgical excision with scalp flap reconstruction. The lesion was analyzed 3 times for pathologic diagnosis; however, none of the prior specimens had undergone immunostaining. He was subsequently diagnosed with mixed desmoplastic melanoma, at least 6.84 mm in depth.

## QUESTIONS

1. What is desmoplastic melanoma (DM)?
2. What are some of the proteins involved in DM expression and diagnosis?
3. What is the preferred management for DM?
4. What is the prognosis for patients with DM?

## DISCUSSION

Desmoplasia is the growth of fibrous or connective tissue. The lesions of DM appear clinically benign, arising from plaques or nodules that can subsequently develop into a fibrous, subcutaneous tumor deep into the primary lesion. They are often misdiagnosed and receive inadequate and sometimes provocative treatments due to their inconspicuous presentation.^1^^,^^2^ Desmoplastic melanoma can present with or without pigmentation; men are affected twice as often as women, and it most commonly affects older patients. The 3 most common locations for DM include head and neck (53%), extremities (26%), and trunk (20%).^3^ The common differential includes neurofibroma, spindle cell sarcoma, schwannoma, dermatofibroma, blue nevus, fibromatosis, and scar.^4^ Desmoplastic melanoma has a propensity for neurotropism, frequently associated with nerve invasion, and spread along fascial planes.^3^ Since most of these tumors are located in the head and neck, this can lead to cranial neuropathies.^5^ Histologically, the lesions have dermal and subcutaneous spindle-shaped cells arranged as a single infiltrate or organized into fascicles.^3^

Proteins are downstream of upregulated genes. Identification of specific proteins associated with melanoma progression may provide prognostic indicators and therapeutic targets. Desmoplastic melanoma and superficial spreading melanoma (SSM) have variably expressed proteins. Desmoglein 1 is one protein expressed higher during the development of DM than SSM. Heat shock proteins (HSPs) are uniformly elevated in SSM in comparison with DM. HSPs have been postulated to protect tumor cells from destruction by innate immunity, promote cell-cycle dysregulation, invasion, and neovascularization.^6^ Immunohistochemically, the tumor cells of DM often fail to react with many antibodies such as melan-A but are usually positive for S100 protein, nerve growth factor receptor, and SOX10 gene. Neurofibromin 1 is the gene most commonly mutated in DM, 93% of the time, usually resulting in nonfunctional proteins.^7^ SOX10 protein is a transcription factor important for neural crest, peripheral nervous system, and melanocytic development. SOX10 is highly specific and sensitive for malignant melanoma, including DM and spindle cell melanomas, being expressed 98% of the time.^8^

Surgical excision is the current treatment of choice; yet, there have not been established optimal margins. Because of the depth of invasion at the time of diagnosis, achieving clear surgical margins upon extirpation becomes difficult. This is especially true in large resections of aesthetically sensitive areas, such as the head and neck. Low incidence of lymph node involvement, ranging from 4% to 14%, distinguishes it from other types of melanoma.^4^ Low incidence of regional lymph node metastases suggests that elective lymph node dissection is not indicated.^3^ There may be benefit to identifying, and histologically evaluating, nerves encountered during the resection.^5^ In patients with positive surgical margins, one study showed a local recurrence rate of 14% in radiotherapy patients as compared with 54% in those who did not. Thus, evidence shows adjuvant radiotherapy should be the standard treatment of DM patients with positive margins, advanced Clark level, Breslow thickness 4 mm or greater, recurrent DM, inoperable DM, and DM with neurotropism (DNM).^4^

Patients with DM had slightly better survival outcome than other melanomas, with the 5-year survival for DM at 75% and 5-year disease-free survival at 62%. With Breslow thickness less than 4 mm, the incidence of local recurrence is close to 20%, whereas among those with Breslow thicknesses above 4 mm, recurrence rates are close to 40%.^3^ Surgical margins less than 1 cm have been associated with significant increases in local recurrence as well as a decreased 5-year overall survival.^4^ Neurotropism is thought to significantly worsen the prognosis of DM by causing deeper infiltration into soft tissues and extending along peripheral nerves. One study showed a statistically significant difference in survival between patients with DM and patients with DNM, with 8-year survival of 90% for patients with DM compared with 60% for patients with DNM.^5^

## Figures and Tables

**Figure 1:**
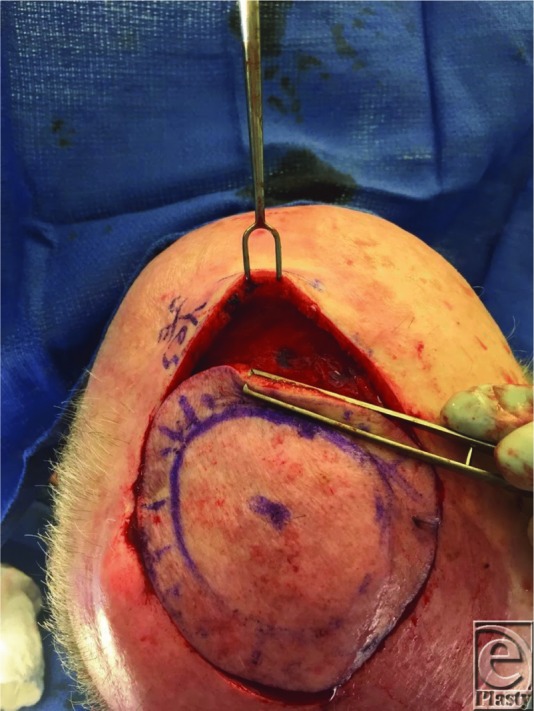


**Figure 2:**